# Reoperation 10 years after Nuss procedure failed: Wung procedure combined with Wenlin procedure

**DOI:** 10.1093/jscr/rjac545

**Published:** 2022-12-05

**Authors:** Wenlin Wang, Weiguang Long, Yang Liu, Bin Cai, Juan Luo

**Affiliations:** Department of Chest Wall Surgery, Guangdong Second Provincial General Hospital, Guangzhou, China; Department of Chest Wall Surgery, Guangdong Second Provincial General Hospital, Guangzhou, China; Department of Chest Wall Surgery, Guangdong Second Provincial General Hospital, Guangzhou, China; Department of Chest Wall Surgery, Guangdong Second Provincial General Hospital, Guangzhou, China; Department of Chest Wall Surgery, Guangdong Second Provincial General Hospital, Guangzhou, China

## Abstract

After the failure of Nuss procedure for pectus excavatum, the risk of reoperation is high, and it is difficult and challenging. Recently, we performed the reoperation for a patient who had failed Nuss procedure 10 years ago. During the operation, Wung procedure and Wenlin procedure were combined to be used and satisfactory results were obtained.

## INTRODUCTION

Nuss procedure is commonly used to treat pectus excavatum [1]. However, there are often cases of surgical failure [2, 3]. Because these patients have serious adhesion behind the sternum, if Nuss procedure is used again, it is not only difficult but also risky [2, 3]. The most dangerous event is heart injury. Once such an event occurs, it will lead to fatal consequences [3]. Therefore, Nuss procedure is not an appropriate choice for patients who need to be operated again. Recently, we performed a reoperation on a patient who failed to Nuss procedure 10 years ago. We performed Wung procedure [4] and Wenlin procedure [5, 6] in the operation, and achieved satisfactory results.

## CASE REPORT

**Figure 1:**
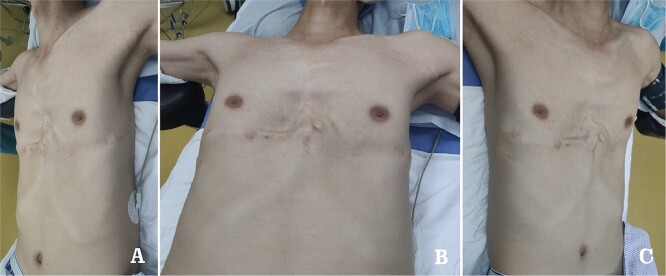
Appearance of chest wall before operation. (**A**) Left side view; (**B**) front view; and (**C**) right side view.

**Figure 2:**
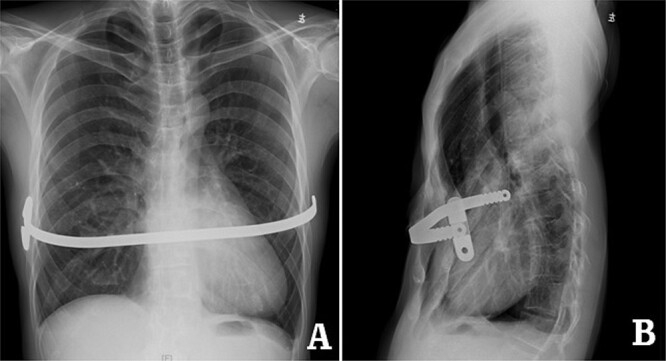
X-ray examination before operation. (**A**) Posteroanterior radiograph; and (**B**) lateral radiograph.

**Figure 3:**
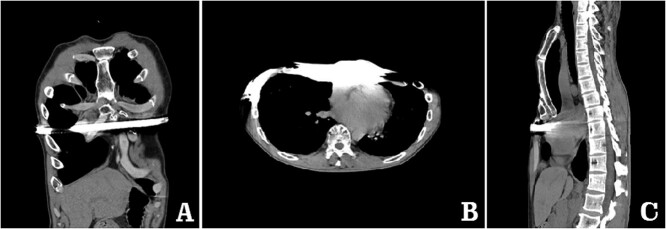
Computed tomography examination before operation. (**A**) Coronal view; (**B**) sectional view; and (**C**) sagittal view.

**Figure 4:**
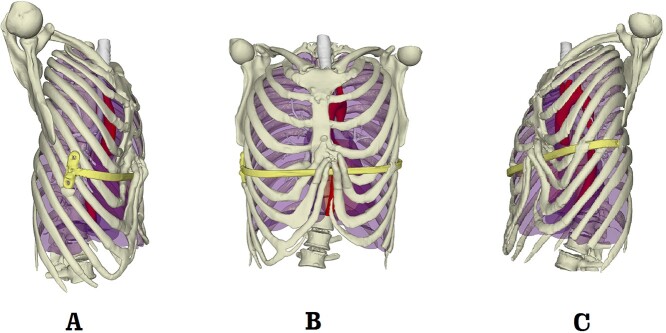
Preoperative 3D reconstruction pictures. (**A**) Right side view; (**B**) front view; and (**C**) left side view.

**Figure 5:**
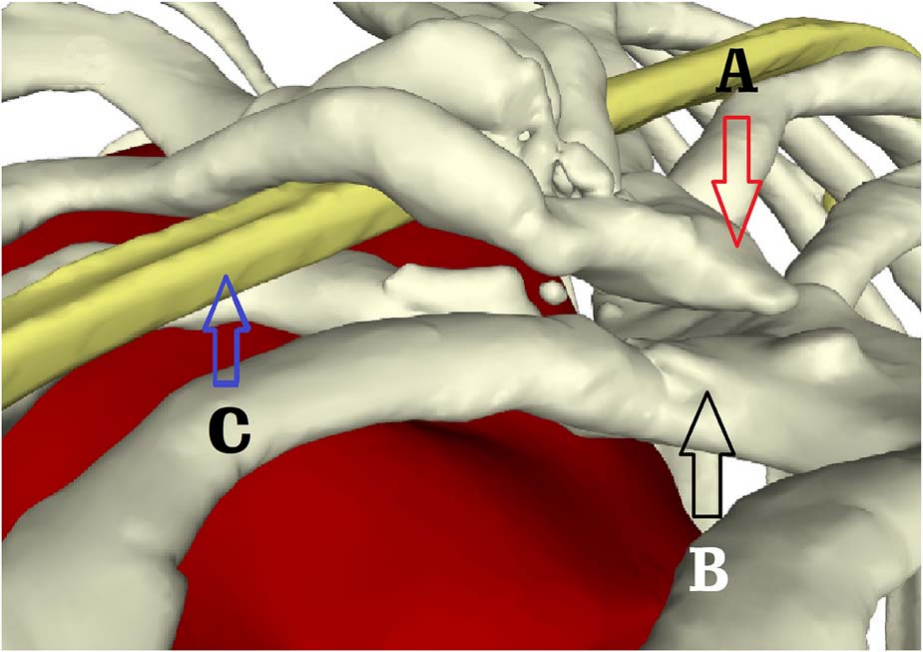
Partial view of the lower sternum. (**A**) End of the severed sternum; (**B**) lower end of sternal body and (**C**) steel bar of the first operation.

**Figure 6:**
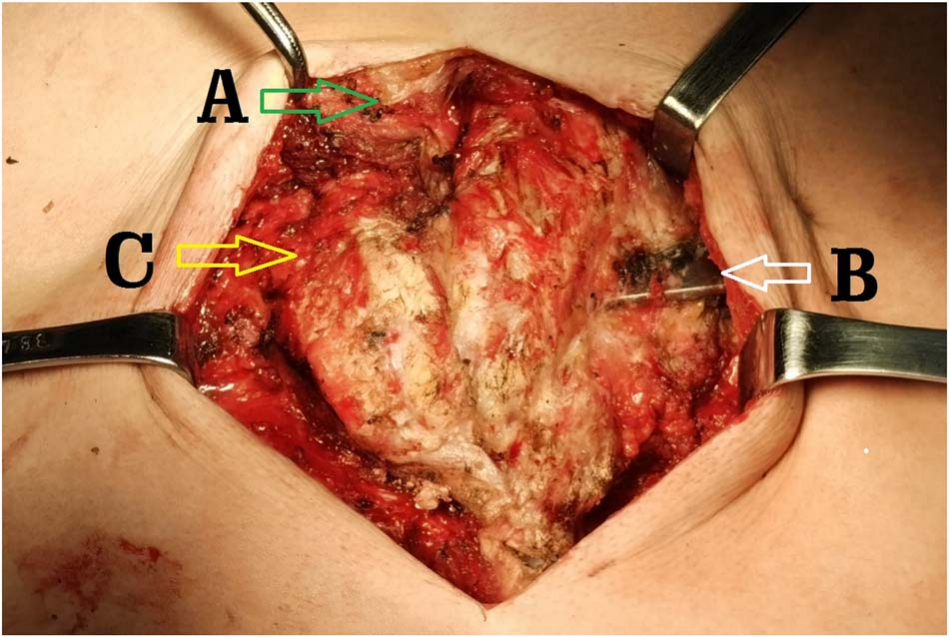
Operative field through the median incision. (**A**) Lower end of sternal body; (**B**) steel bar of the first operation and (**C**) end of sternum and rib arch supported by the steel bar.

**Figure 7:**
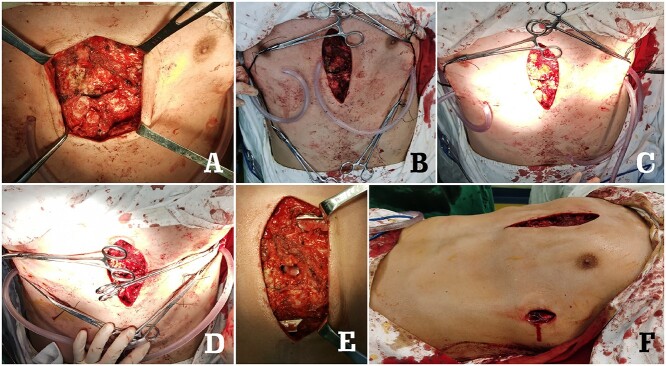
Surgical pictures. (**A**) The anterior chest wall structures; (**B**) and (**C**) Wung procedure was performed; (**D**) Wenlin procedure was performed; (**E**) median operative field after Wung procedure and Wenlin procedure and (**F**) appearance of chest wall before incisions sutured.

**Figure 8:**
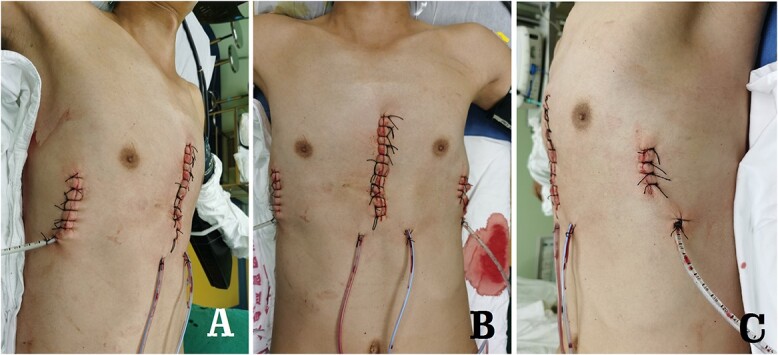
Postoperative appearance of chest wall. (**A**) Right side view; (**B**) front view and (**C**) left side view.

**Figure 9:**
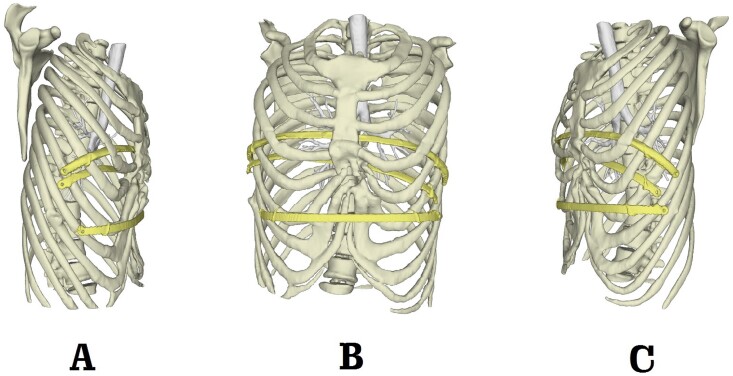
Postoperative 3D reconstruction pictures. (**A**) Right side view; (**B**) front view and (**C**) left side view.

The patient was a 32-year-old male who underwent a Nuss procedure for pectus excavatum in the local hospital 10 years ago. The appearance of his anterior chest wall was slightly improved after the operation, but the depression did not disappear. Since then,  the  depression  has  continued  to  increase.  Recently,  the

patient suffered from chest pain and dyspnea, and was admitted to our hospital for surgical treatment. Preoperative physical

examination showed that the anterior chest wall was sunken and uneven in the middle, and two scars were visible on the lateral chest wall (Fig. 1). The preoperative imaging examination showed that there was a steel bar in the chest wall, and the anterior chest wall was depressed. The lower part of the sternum was broken, with its distal end was supported up by the steel bar. The lower end of the sternum was located deep beneath the steel bar, and the heart was obviously compressed (Figs 2–5). The operation was performed under general anesthesia. Incisions were made in the middle of the depression and two sides of chest wall. After the steel bar was taken out, the adhesion behind the sternum was separated through the median incision. Wung procedure was performed with two steel bars to support the depressed lower part of sternum [4], and then, the third steel bar was used to perform Wenlin procedure to flatten the protrusion part of the rib arch [5, 6] (Figs 6 and 7). During the two procedures, Wang technique was used to fix all the steel bars [7]. After the two procedures were completed, drainage tubes were placed in bilateral thoracic cavities and median surgical field, and the incision was closed to end the operation (Fig. 8). The operation time was 90 min, and the operation was smooth without any complications. The appearance of chest wall returned to normal after the operation, and imaging examination showed that the position of steel bars was satisfactory (Figs 8 and 9). The patient was discharged 7 days after operation.

## DISCUSSION

There are many surgical methods for pectus excavatum, and Nuss procedure is the most popular one [1, 4, 8]. This procedure is a minimally invasive surgery with many advantages [1, 2]. However, if this procedure is not operated properly, complications and even failure may occur [2, 3]. For the operation failed patient, the depression of the anterior chest wall will recur, which generally requires another operation. Since there is serious adhesion behind the sternum after the first operation, if Nuss procedure is used again, the operation will be difficult and dangerous. Therefore, this procedure is not an ideal choice for reoperation after the failure of the first Nuss procedure [3].

Wung procedure is a modified Nuss procedure, and its operation details are almost completely different from the standard Nuss procedure [4]. Its biggest advantage is safety and simplicity. In the reoperation after the failure of Nuss procedure, Wung procedure can generally obtain satisfactory results. This patient experienced a failed Nuss procedure 10 years ago, and the anterior chest wall depression recurred. Since Nuss procedure was not suitable for reuse, Wung procedure became the first choice. In this reoperation, we used two steel bars to support the depression, thus achieving the purpose of Wung procedure.

After the depression was eliminated, the rib arch was obviously raised and needed to be corrected. We chose Wenlin procedure for correction. Wenlin procedure is a technique designed for protrusions [5, 6], which is a typical template plastic surgery [9]. We used the third steel bar for this procedure and obtained satisfactory results.

The reoperation after the failure of Nuss procedure is a great challenge, which requires not only good surgical concepts, but also appropriate surgical methods. Our experience shows that Wung procedure combined with Wenlin procedure is a safe and simple choice. However, as the deformities caused by the failure of Nuss procedure may be very complex, it is necessary to make appropriate choices according to the specific characteristics of the deformities.

## CONFLICT OF INTEREST STATEMENT

None declared.

## FUNDING

None.
